# Research Status in Clinical Practice Regarding Pediatric and Adolescent Bipolar Disorders

**DOI:** 10.3389/fpsyt.2022.882616

**Published:** 2022-05-27

**Authors:** Lu Liu, Ming Meng, Xiaotong Zhu, Gang Zhu

**Affiliations:** ^1^Department of Psychiatry, The First Affiliated Hospital of China Medical University, Shenyang, China; ^2^Department of Psychiatry, The Fourth Affiliated Hospital of China Medical University, Shenyang, China; ^3^Shenyang Mental Health Center, Shenyang, China

**Keywords:** adolescent bipolar disorder, comorbidity, pediatric bipolar disorder, epidemiology, medication

## Abstract

Bipolar disorders (BDs) have high morbidity. The first onset of 27.7% of BDs occurs in children under 13 years and of 37.6% occurs in adolescents between 13 and 18 years. However, not all of the pediatric and adolescent patients with BD receive therapy in time. Therefore, studies about pediatric and adolescent patients with disorders have aroused increased attention in the scientific community. Pediatric and adolescent patients with BD present with a high prevalence rate (0.9–3.9%), and the pathogenic factors are mostly due to genetics and the environment; however, the pathological mechanisms remain unclear. Pediatric and adolescent patients with BD manifest differently from adults with BDs and the use of scales can be helpful for diagnosis and treatment evaluation. Pediatric and adolescent patients with BDs have been confirmed to have a high comorbidity rate with many other kinds of disorders. Both medication and psychological therapies have been shown to be safe and efficient methods for the treatment of BD. This review summarizes the research status related to the epidemiology, pathogenic factors, clinical manifestations, comorbidities, diagnostic and treatment scales, medications, and psychological therapies associated with BDs.

## Introduction

In recent years, as high morbidity of (BD) has been reported, increasing attention has been focused on this field, especially in pediatric and adolescent patients with BD. However, not all of pediatric and adolescent patients with BD receive therapy in time. This may be due to the lack of adequate knowledge about this field. Therefore, this narrative review attempted to summarize the research progress from 1998 to 2021 related to the epidemiology, pathogenic factors, clinical manifestations, comorbidities, diagnostic and treatment scales, medications, and psychological therapies associated with BDs. The pathogenic factors are mostly due to genetics and environment; however, the pathological mechanisms remain unclear. Pediatric and adolescent patients with BDs manifest differently from adult patients with BDs and the use of scales can be helpful for the diagnosis and the treatment evaluation. Pediatric and adolescent patients with BDs have been confirmed to have a high comorbidity rate with many other kinds of disorders. Both medication and psychological therapies are safe and efficient methods for the treatment of BD. We aimed to introduce the basic concept and related research results and produce a new therapy thought for pediatric and adolescent patients with BD in the future.

## Epidemiology

In recent times, bipolar disorder (BD) has high morbidity worldwide. Merikangas et al. (1) stated that the lifetime prevalence rate of BD was 0.6%, BD type II was 0.4%, subthreshold BD was 1.4%, and BD type I and subthreshold BD was higher in boys than girls, with girls having a higher prevalence of BD type II. The 12-month prevalence rate of BD in Australia was 0.9%, lower than that of the United States (2.8%) and New Zealand (2.2%), but similar to the global rate (1.5%) (2). However, half of the BD type I and subthreshold BD cases manifest before the age of 25 (1); the first onset of symptoms in 27.7% of individuals is before the age of 13, with 37.6% having initial symptoms between 13 and 18 years old (3). A meta-analysis by Van Meter et al. (4) showed that the average prevalence rate of pediatric BD in the United Stated and Europe was 3.9% while that of pediatric BD type II was 0.6%. A meta-analysis that included studies from 1985 to 2007 stated that the total prevalence rate of pediatric BD was 1.8% (5). The high morbidity of pediatric and adolescent patients with BD is thus a concern. Meanwhile, its early onset is associated with a higher comorbidity rate with anxiety disorder and substance abuse disorder and presents a higher recurrence rate, shorter interval, and higher possibility of suicide and violence (3).

Unfortunately, not all of the pediatric and adolescent patients with BD receive timely therapy. A meta-analysis of Cervesi et al. (6) indicated that, among the children who received anti-psychotic treatment, only 13.6% were patients with BD, and only 44% of children with BD received anti-psychotic treatment. Dagani et al. (7) stated that the average time from the onset of BD to receiving therapy was 5.8 years. The delayed response may be associated with delayed diagnosis, which is likely due to the low degree of emphasis on pediatric and adolescent patients with BD, gender differences, and inconspicuous symptoms. Coville et al. (8) demonstrated that the lifetime onset time of mood disorders was longer in boys than girls, with the manifestation of symptoms beginning in boys (71.4%) during childhood (before 13 years of age), whereas, in girls (73.9%), the initial symptoms occurred during puberty. Bipolar disorder occurring during childhood has a longer lifetime onset time than BD that occurred at puberty (8). Boys with BD presented more with concurrent attention deficit hyperactivity disorder (ADHD), whereas girls presented more with concurrent severe depression symptoms; boys usually present with more extrinsic symptoms such as aggressiveness, whereas girls present with more intrinsic symptoms such as depression or anxiety, which may lead to a delayed diagnosis to puberty in girls (8).

## Pathogenic Factors

### Genetics

Bipolar disorder shows prominent familial inheritance. There is an increasing prevalence rate for the offspring of patients that have BD (9). A meta-analysis by Lau et al. (10) indicated that the morbidity of the offspring of at least one of the parents that has BD presented 9 times higher than healthy parents. Geller et al. (11) stated that, for those patients with both BD and substance abuse disorder, 96% had an occurrence of multi-generational mood disorder and 56% had multi-generational substance abuse disorder. A meta-analysis by Wozniak et al. (12) showed that the morbidity of the first-degree relatives of children with BD was higher than that of children with ADHD or healthy children. Biederman et al. indicated that, whether BD and ADHD were comorbid in children or not, there was prominent familial inheritance. The evidence suggested that the morbidity of parents of children only with BD was similar to that of children with both BD and ADHD but higher than that of children only with ADHD or healthy children (13). Furthermore, for children with both BD and ADHD, the morbidity of the parents that had both BD and ADHD was 46% and the morbidity of the parents that only had ADHD was 20%, which meant the comorbidity of BD with ADHD also has familial inheritance (13).

Bipolar disorder has also been confirmed to be related to several types of gene polymorphisms. Stahl et al. (14) confirmed the effectiveness of 11 kinds of locus polymorphisms through a genome-wide association study, with the tetratricopeptide repeat and ankyrin repeat containing 1 locus (TRANK1) presenting the most prominent differences. A meta-analysis by Nassan et al. showed that muskelin (MKLN1) was related with early-onset BD (in patients ≤ 19 years) and that MKLN1 mediated cellular trafficking of the gamma-aminobutyric acid-A (GABA-A) receptor, which indicated that GABA-A may be associated with early-onset BD. The authors then proposed that the low expression of MKLN1 in patients with BD increased GABA-A receptors in the hippocampus, increasing the GABA excitability of young neurons in the early phase, which resulted in a higher risk of mania or hypomania (15). A meta-analysis by Wu et al. (16) confirmed that a single nucleotide polymorphism rs11127876 of cell adhesion molecule 2 was related with early-onset BD (in patients ≤ 20 years old) in the Han population. Ferreira et al. (17) discussed whether the serotonin transporter (5-HT) promoter region's gene polymorphism was related to mania symptoms after the usage of antidepressants among adult patients with BD and found no relationship when antidepressants were used in combination with mood stabilizers; however, when used alone, short allele transformations were more frequently, which manifested in mania or hypomania. However, in pediatric BD, this conclusion has not yet been confirmed. Baumer et al. (18) did found no relationship between 5-HT transporter gene polymorphisms and mania symptoms after the usage of antidepressants among pediatric patients with BD. van Hulzen et al. (19) indicated that patients with BD and ADHD presented with centrosomal protein 85 kDa-like gene (*CEP85L*; chromosome 6) and TATA box binding protein (TBP)-associated factor, 31 kDa pseudogene 2 gene (*TAF9BP2*; chromosome 10) polymorphisms. When the age of the patients was restricted to the early-onset type (i.e., age ≤ 21 years), patients with BD and ADHD presented with adenylate csyclase 2 (*ADCY2*; chromosome 5) polymorphisms (19). Although the number of studies regarding pediatric and adolescent patients with BD gene polymorphisms is growing, there is still a shortage compared with the studies of adult patients with BD. Moreover, most of the study results of adult patients with BD were different from that of pediatric and adolescent patients with BD.

### Neuroimaging Findings

The imaging mechanism of BD is still not clear, but many studies confirmed that BD might be related to neuroanatomical changes, distinct alterations in brain activity, and abnormal metabolism. Lei et al. (20) studied the local anatomical traits of children with BD and found that they presented with an altered structural connector index (increasing cluster coefficient and characteristic path length), which normalized after treatment (lithium and quetiapine). Further, the authors found that the gray matter morphology index was 80% accurate in identifying the response of those children who had received treatment (20). They also showed that prior to treatment, there were node center changes in many brain areas including the right inferior frontal gyrus, bilateral insula, and bilateral supplementary motor area, which meant that the core area of the neocortical anatomical network that mediated emotional processing was decreased; however, after the treatment, node center of the insula, which is closely related to mania symptoms, returned to normal (20). Taken the data together indicated that BD resulted in both total and node structural network changes, which decreased after the treatment, indicating that the therapy efficiency could be predicted and the similar connector normalization response after two kinds of treatments meant the connector change was the downstream effects of related clinical effects of these two kinds of treatments (20). Beyer et al. (21) indicated that patients with BD showed high tensity of deep white matter of the subcortex compared with healthy subjects and that this difference was more prominent in children and adolescent patients with BD. Lu et al. (22) observed a shrunken left dorsalmedial prefrontal lobe and enlarged left putamen in adults with BD, a shrunken right temporal gyrus and increased left precuneus in those with BD type I, and a shrunken orbitofrontal cortex, right claustrum, and right dorsolateral prefrontal cortex in adolescents with BD, all of which illustrated the pathophysiological basis of BD types and indicated significant differences between adult BD and adolescent BD. Lee et al. (23) observed increased activation of the inferior frontal gyrus and low activation of the limbic region during attention studies in children with BD and a lack of activity in the dorsal attention system such as frontal striatum circuit (dorsolateral prefrontal cortex, anterior cingulate gyrus, right lentiform nucleus, and right globus pallidus) that was not evident in adults with BD. This kind of activation differences was also related with drug response, Chang et al. (24) showed that children with BD with decreased activation levels in the prefrontal cortex and increased activation levels in the left ventrolateral prefrontal cortex at base line reacted better to quetiapine.

Hafeman et al. (25) indicated positive functional connectivity between the amygdala and the ventral prefrontal cortex during emotional processing in adolescents with BD, which was only found in cases of BD. The results indicated that, besides the cortex, abnormalities in the amygdala abnormality were also directly related to BD. Hajek et al. (26) illustrated that the volume of the left amygdala was prominently smaller in children with BD compared with normal children, whereas a larger volume was found in adults with BD compared with normal adults. The results indicate that a shrunken amygdala may be a vulnerability marker associated with BD and that the increased volume later in life may be due to recurrent mood disorders or drug effects as antidepressants, such as lithium, can increase the volume of the hippocampus and the amygdala (26). Another possibility may be children with BD mostly presenting with vulnerable emotional states such as irritability, impulsiveness, and attention deficits, which is different from adults with BD; some other possibilities could be that the adolescents with BD were more likely to have ADHD and that patients with ADHD had smaller amygdalar volumes (26). A meta-analysis by Pfeifer et al. (27) also supported the above results, where children with BDP presented with smaller amygdalar volumes compared with healthy subjects, a feature that was not prominent in adults, regardless of BD diagnosis.

A meta-analysis by Gigante et al. (28) summarized studies from 1980 to 2010 and they stated that, in both adults and children with BD, brain-derived glutamic acid levels were higher than that of healthy subjects. Chang et al. (29) observed a decreased *n*-acetylaspartate level in the dorsolateral prefrontal cortex in children with BD, which is similar to the previous results obtained from the studies on adults with BD. Taken together, the data suggest that the altered *n*-acetylaspartate levels might be a pathological mechanism of BD and that the low levels of *n*-acetylaspartate predict decreased nerve density and developmental capacity (29). Gracious et al. (30) showed that linseed oil could improve the disease severity of pediatric and adolescent patients with BD by increasing serum eicosapentaenoic acid levels and decreasing arachidonic and docosapentaenoic acid levels.

### Other Factors

Other factors that are known to affect pediatric and adolescent patients with BD are related to environmental factors, a history of abuse, life events, and family relationships. Blais et al. (31) stated that those patients with BD that had a history of abuse before 18 years of age presented with more severe manic/depressive symptoms and psychosis, had higher levels of comorbidity with post-trauma stress disorder, anxiety disorder, substance abuse disorder, and alcohol abuse disorder, and were more prone to the early onset of BD and higher risk of rapid cycling, frequent mania episodes, frequent depression episodes, and risk of suicide. Tillman et al. (32) assessed pediatric and adolescent patients with BD with a life event checklist and divided the list into dependent events (such as deaths), independent events (such as failure in academia), and non-specific events (such as a partner going to school abroad, which is not necessarily related to the disease) and found that dependent, independent, and non-specific events were more frequent in pediatric and adolescent patients with BD than in patients with ADHD and healthy subjects. Further, patients with BD had significantly more dependent events than patients with ADHD, which meant that the dependent events might be one of the reasons for the onset of BD (32). Coville et al. (8) indicated that highly expressed emotional states exhibited by parents of children with BD increased the possibility of recurrence, which was unrelated to the severity of the disease; in particular, parents of girls with BD were more likely to begin to express criticism of the child during puberty whereas parents of boys began to express criticism of the child at an earlier stage. Sullivan et al. (33) studied family traits of adolescent patients with BD and discovered that their families presented more impaired intimacy and adaptability and conflicts than the families of healthy subjects, and the highly emotional parents showed lower intimacy and adaptability and more conflicts. Further, parents who expressed more criticism generate more conflicts with their offspring, which indicated that family therapy to early-onset BD needs to focus on family intimacy, adaptability, and levels of conflicts (33). Moreover, family relationships and life events can also affect the prognosis of BD. Kim et al. (34) illustrated that chronic stress in the family unit and in intimate and peer relationships were related to the poor improvement of affective symptoms in children with mood disorders. Further, the frequency of dependent events was also related to poor improvement of affective symptoms, which was more prominent in older adolescents (34). Finally, chronic stress in intimate relationships predicted poor improvement of depression and complex affective symptoms and was more likely to predict complex affective symptoms with age, whereas chronic stress in peers relationships predicted poor improvement of mania symptoms (34).

Bipolar disorder developed from subthreshold BD should also be paid attention to. A meta-analysis by Vaudreuil et al. (35) demonstrated that, compared with healthy subjects, children with subthreshold BD showed more severe functional impairment and higher rates of disruptive behaviors, mood disorders, substance abuse disorders, and suicidal ideation. Compared with subthreshold BD, children with BD type I appeared to have a more severe functional impairment and severe mania symptoms and higher rates of suicidal ideation and attempts and mental health therapy session; however, the severity of depression symptoms or comorbidity with other disorders, was similar between the patients with BD and the patients with subthreshold BD, which meant that the subthreshold BD is likely associated with a pathological state that needs clinical attention and treatment (35). Ratheesh et al. (36) stated that 4 of 100 major patients with depression disorder converted to BD within 2 years and that a quarter of major depression disorder adults and adolescents would get converted to BD after 12–18 years. The study indicated that these results were directly related to family history of BD, earlier onset of depression, and appearance of psychosis symptoms and that subthreshold hypomania symptoms were a predictor of BD conversion, where the onset of depression conversed to BD lately was 4.8 years earlier than those did not converse to BD (36).

## Clinical Manifestation

### Affective Symptoms

Compared with adult patient with BD, pediatric and adolescent patients with BD has various symptoms, and the ratio of irritability is higher than that of euphoria. Tillman et al. (32) indicated that, among pediatric and adolescent patients with BD, 89.3% presented with feelings of elation, 86% with grandiose, 71% with the flight of ideas/racing, 54.8% with mixed manic, 60.3% with psychotic, and lastly, 40.9% without lifetime or current depression onset. The most frequently appearing symptoms were increased energy, attention deficits, and imperative language (37). In total, four-fifth of the children with BD showed increased levels of irritability and grandiosity, over 70% showed euphoric and excited tendencies, decreased sleep patterns, and racing thoughts; 69% had poor judgment; 50% experienced frequent fearful tendencies, and just over a third had erotic tendencies and psychosis (37). Wozniak et al. (38) demonstrated that, for abnormal mood in children with BD, the rate of severe irritability (94%) was higher than that of euphoria (51%) and that grandiosity did not frequently appear in children with mania; moreover, neither the rate of grandiosity was unrelated with irritability alone or irritability comorbid with euphoria nor was the manifestation, comorbidity, or function related with euphoria. These findings challenged the view that euphoria was the main symptom of children with mania and emphasized that severe irritability had more universal representation (38). This kind of phenomenon might be related with special affective state in childhood and puberty. The main psychopathological features of child mood disorder are polymorphisms of simultaneous appearance of the following two varieties of affective disorders: Instability that is not fixed to one episode and high occurrence of overvalued ideas reflecting adolescent special affective state accompanied with psychological crisis at puberty (39). Indeed, both of these frequently appeared mixed affective states and the significance of classification of BD indicate the need for special drug or psychological therapies to adolescent BD (39).

Many symptoms of pediatric and adolescent patients with BD are similar with ADHD. Therefore, the classification diagnosis is quite important. Geller et al. (40) stated that the following five factors could differentiate BD from ADHD, Excited, grandiosity, flight of ideas/racing thoughts, decreased sleep, and eroticism, which were also specific diagnostic criteria of mania in The Diagnostic and Statistical Manual of Mental Disorders (DSM-IV), but did not overlap with the diagnosis of ADHD. However, it must be noted that several symptoms/states do overlap, including irritability, increased activity, accelerated speech, and attention deficits, which are observed in children with BD and ADHD, thus having no significant differentiation (40). Moreover, excitement and irritability appear simultaneously in 87.1% of children with BD (40).

### Suicidal Ideation

Suicide is a major risk factor in pediatric and adolescent patients with BD. Crescenzo et al. (41) indicated that the suicide rate of adolescents with BD (31.5%) was higher than that of those with major depression disorder (20.5%), which was higher than that of those with mania or hypomania (8.49%). Tillman et al. (32) demonstrated that 24.7% of pediatric and adolescent patients with BD exhibited suicidal tendencies. Stanley et al. (42) showed an increased suicide risk in 57% of children with BD. Weinstein et al. (43) showed that 53.5% of children with BD reported long-term suicidal ideation, 39.4% reported transient suicidal ideation, 23.9% reported a history of severe suicidal ideation (having a specific plan), 14.1% reported undertaking severe suicidal ideation, 35.2% reported history of suicide behavior (the act of preparation, the act of being prevented or occurring), and 2.8% reported recent suicidal behavior; however after treatment, none reported suicidal behavior. Thorell et al. (44) showed that the reduced electrodermal activity was specifically related to suicidal ideation and that the suicide rate was highest in adolescents with BD and was unrelated to the severity of depression, anxiety type, gender, or age, indicating that the reduced electrochemical activity was a possible marker of suicide ideation.

### Sleep

Children with BD exhibit a decreased need for sleep and changes in sleep rhythms. Tillman et al. (32) stated that 39.8% of pediatric and adolescent patients with BD exhibited a decreased need for sleep. Lunsford–Avery et al. (45) indicated that adolescent patients with BD who presented with sleep problems, i.e., stable sleep rhythms were related to decreased mania scores, unstable morning routines, and difficulty in waking up were related to increased mania scores. The study also showed that moderate insomnia and an increased number of nocturnal awakenings were related to increased depression scores, which meant that sleep impairment was related to mania and depressive symptom severity in patients with BD and that sleep/awakening rhythms had a protective effect (45).

### Cognitive Impairment

Pediatric and adolescent patients with BD may lead to cognitive impairment. Research has indicated that children with BD exhibit an impairment in language learning (46, 47), language memory (46–48), working memory (46), visual learning (46), visual memory (46, 48), processing speed and attention maintenance (47, 48), and accuracy of emotional adjustment tasks (especially at facial emotion recognition and emotion language interference tasks induced by cognition) (49). However, it must be noted that the data regarding alterations in working memory remain controversial. A meta-analysis by Elias et al. (46) indicated that there was impairment in working memory in children with BD but Bora et al. (48) found no differences in planning or working memory in children with and without BD. Joseph et al. showed that, compared with healthy subjects, the most significant difference in children with BD was language memory, and moderate differences in attention, executive function, working memory, visual memory, visual perceptional ability, and verbal fluency were observed. It should be noted that small differences were found in reading processing speed abilities and total IQ levels (50). Halac et al. (51) also demonstrated that children with BD presented with significantly worse social cognitive ability and emotional cognitive impairment even in euthymia, which was unrelated to age, gender, affective symptom severity, IQ level, comorbidity with ADHD, or drug treatment. Nieto et al. (47) stated that the cognitive impairment in pediatric and adolescent patients with BD (such as language learning and memory, processing speed, and executive control) was similar but less severe than early-onset schizophrenia, which pointed to a mental disease continuous spectrum or represented some degree of genetic biooverlap.

### Other Symptoms

Mick et al. (52) used a child behavior list and showed that scores of aggressivity, attention, and anxiety depression subscale scores were above 70. Klages et al. (53) indicated that, compared with healthy subjects, pediatric and adolescent patients with BD exhibited increased encopresis (15.1 vs. 3.2%) and enuresis (21.5 vs. 6.4%), that all enuresis appeared in patients without lithium treatment; most of encopresis and enuresis appeared before mania, and that the hostility of the mother toward children with encopresis was greater than toward children without encopresis.

## Diagnosis and Efficient Assessment Scales

Besides definite diagnosis criteria, the usage of some clinical scales is also beneficial to help diagnoses. Kahana et al. (54) indicated that the reporting by parents had more significance for diagnosis than reporting by children themselves or teachers. Indeed, the efficiency of the Children Behavior Check List (CBCL) was not high and was likely related to the paired reports of parent–teacher and parent–children (54). Further, the CBCL does not assess mania or hypomania symptoms or include items for special diagnosis significance such as grandiosity and flight of ideas; therefore, the classification of adolescent BD needs more specific tools such as parent general behavior surveys or the Young Mania Rating Scale (YMRS) (54). Findling et al. (55) found that the mania scores of the parent-rated 10-item General Behavior Inventory Mania (GBI-M10), YMRS, and Clinical Global Impression–Bipolar Disorder (CGI-BD) in children with BD were similar, but the line item effect sizes of the subject-rated GBI-M10 were smaller, which meant the parent-rated GBI-M10 had higher accuracy to detect the efficiency of treatment outcome. Youngstrom et al. (56) showed that, in assessment scales of clinical total symptom improvement, the accuracy order from the highest to the lowest was ≥ 50% reduction in YMRS scores, combined scores [YMRS <12.5, Children Depression Rating Scale-Revised (CDRS-R) ≥ 40 and Child Global Assessment Scale (CGAS) ≥ 50], 95% reliable change of CGAS, ≥ 30% reduction of YMRS scores.

## Comorbidity

Three-quarters of BD cases are comorbid with at least one other kind of disorder including anxiety disorder, especially panic disorder as the most comorbid disorder, behavior disorder (44.8%), substance abuse disorder (36.6%), and BD type I (88.2%) and type II (83.1%), which were higher than subthreshold BD (69.1%) (1). This kind of comorbidity rate also existed in pediatric and adolescent patients with BD. Eser et al. (57) indicated that the comorbidity rate of children with BD with any kind of anxiety disorder was 44.7%, panic disorders was 12.7%, generalized anxiety disorder was 27.4%, social phobia was 20.1%, separation anxiety disorder was 26.1%, and obsessive–compulsive disorder was 16.7%. Children with BD were likely to be comorbid with generalized anxiety and separation anxiety disorders, whereas adolescents with BD were more likely to present with panic disorder, obsessive–compulsive disorder, and social phobia (57). Stanley et al. (42) discovered that 14% of children with BD had a concurrent diagnosis of transient anxiety disorder. Weintraub et al. (58) stated that adolescent BD with anxiety resulted in longer depression outcomes and more severe hypomania symptoms and family conflicts, whereas the manifestation of adolescent BD with ADHD resulted in longer hypomania outcomes, slower improvement of hypomania symptoms (the family-focused therapy was more efficient than psycho-education enhancement care), and more family conflicts. Weinstein et al. (43) demonstrated that the comorbidity rate of children with BD and ADHD was 79%, the oppositional defiant disorder was 37%, anxiety was 36%, and behavior disorder was 9%. Tillman et al. (32) found that 87.1% of pediatric and adolescent patients with BD cases were comorbid with ADHD. Geller et al. (11) confirmed that, for BD with substance abuse disorder, the most frequent type was alcohol and cannabis. Amerio et al. (59) showed that the comorbidity rate of pediatric and adolescent patients with BD with obsessive–compulsive disorder was 24.2%, which was higher than that found in the adult patients with BD (13.5%). Stanley et al. (42) indicated that 25.3% of children with BD had confirmed DSM-IV diagnoses of corresponding nightmares, 4.5% had night terrors, 7.1% had sleep-walked, in terms of permanent sleep disorder, 35.9% had nightmares, 0.8% corresponded with a night terror, and 12.9% corresponded with sleep-walking. The study stated that children with BD with nightmare had 2 times greater risk of suicide, which was still prominent after excluding trauma history, anxiety, and depression symptoms; however, for children with BD with night terror or sleep-walking, there was no increase in suicide risk (42).

## Medication

### Mood Stabilizers

Lithium and valproate have been confirmed to be effective in the treatment of pediatric and adolescent patients with BD. Patel et al. (60) administered lithium therapy (30 mg/kg) to adolescent patients with BD type I episodes, monitored plasma concentrations 1.0–1.2 mEq/L for 6 weeks, and found that the response and remission rates were, respectively, 48 and 30%, with only mild and moderate side effects that included headache (74%), nausea/vomiting (67%), stomach ache (30%), and abdominal cramps (19%). Moreover, when the therapy was extended to 28 weeks, the benefits remained and there were little changes in the severity of side effects (61). Findling et al. (62) administered 16 weeks of additional lithium treatment after and an initial 8-week period to children with BD type I and found that the response rate was 68.3% in the final 8 weeks, which was higher than the response rate after 8 weeks of treatment, and the remission rate was 53.7%, with only side effects including nausea, headache, abdominal pain, and tremors. Cipriani et al. (63) stated that, compared with placebo, lithium decreased suicidal ideation in patients with mood disorder, although having no significant effects on self-injury but still better than carbamazepine. Geller et al. (11) confirmed that lithium was effective in BD and secondary substance abuse disorder. Abnormalities in the phosphoinositol signaling pathway are considered a neurophysiological mechanism of BD; therefore, it was considered that the effectiveness of lithium in reducing BD symptoms was related to the degradation of neuroinositol *via* inhibition of monophosphatase (64). However, Patel et al. (64) measured inositol content in the medial, the left lateral prefrontal cortex, and the right lateral prefrontal cortex after administration of lithium to depressed children with BD and found no differences in baseline content before or after treatment, which indicated that inositol degradation was not the mechanism that mediated the effects of lithium treatment.

Barzman et al. (65) administered divalproex sodium to children with BD comorbid with disruptive behavior disorder for 28 days (plasma concentration 80–120 μg/ml) and found that it was effective in reducing impulsive and aggressive behaviors, so divalproex sodium could be used as a monotherapy for impulsion and responsive aggressive behavior. Scheffer et al. (66) administered sodium valproate to children with BD who are comorbid with ADHD for 8 weeks and found that 80% of children had ≥ 50% reduction in YMRS scores, whereas only 7.5% showed improvement of ADHD symptoms. The authors then added mixed amphetamine salts to the basic sodium valproate treatment program for 4 weeks and found that the reduction in ADHD symptoms was 1.9 times higher than the placebo, without any significant side effects or exacerbation of mania, which meant giving mixed amphetamine salts to children with BD comorbid with ADHD after mania symptoms had been controlled after sodium valproate treatment was safe and effective but giving sodium valproate monotherapy was ineffective to ADHD (66).

Lamotrigine has also been confirmed as an effective treatment against BD. Suppes et al. (67) administered lamotrigine (50–600 mg/day) to patients with BD (Including children and adolescents) and found that 65% had significant improvement in clinical global impression scale scores, depression symptoms, and mood cycles, which meant that lamotrigine was effective for mood stability and antidepression treatment. Findling et al. (68) administered BD type I children with lamotrigine as a supplement therapy and found that the efficiency was similar to placebo but that positive effects were more prominent in older adolescent patients (13–17 years old).

Gracious et al. (69) administered lithium and sodium valproate to children with BD for 20 weeks and found that 24.4% had increased thyroid-stimulating hormone (TSH) levels; the higher the TSH levels at baseline and the higher the lithium levels administered, the more increased were the TSH levels at final presentation. This problem reminds us that we should better monitor TSH levels in the usage of mood stabilizers.

### Antidepressants

Considering the risk of conversion to mania episodes, the use of antidepressants to treat patients with BD is cautioned. Ghaemi et al. (70) indicated that, in adult patients with BD in an acute depression phase, the combination of mood stabilizers and citalopram did not affect treatment efficiency or exacerbate mania during acute mania episodes but that the addition of citalopram to rapid-cycling patients with BD exacerbated mania symptoms. Henry et al. discussed risk factors leading BD conversion to hypomnia or mania including age, gender, diagnosis (type I or II), previous mania onset times, and therapy methods (electroconvulsive therapy vs. antidepressants such as selective serotonin reuptake inhibitors, types of mood stabilizer like lithium vs. anticonvulsants) and found that the total conversion rate to hypomania and mania was 27%. The conversion rate under selective serotonin reuptake inhibitors treatment was 24%, and the conversion rate of those under lithium treatment (15%) was significantly lower than without lithium treatment (44%). Gender, age, diagnosis, and previous mania onset times did not affect conversion rates (71). Finally, there were no differences in conversion rates when anticonvulsant therapy was used compared with those without mood stabilizers treatment (71). Offidani et al. (72) indicated that, after antidepressant administration to adolescents with depression or anxiety, the total rate of mania or hypomania was 8.19% (depression was 10.4%, anxiety was 1.98%), which was higher than the patients without drug treatment (0.17%). Further, the patients exhibited overactivated responses (including insomnia, various behavioral excitation response and hyperactivation responses, restlessness, agitation, irritability, persistent anger, hypomania, and mania), depression (11.2%), and anxiety (13.8%) 3–10 times more than patients without treatment (72). Taken together, the data indicate that antidepressant treatment may lead to mood instability in undiagnosed patients with BD and emphasized the necessity for the usage of mood stabilizers (72). Further, the data showed that over-activated responses that also appeared in patients with anxiety indicated that adolescent anxiety disorders might represent an important pathway in the conversion to BD (72).

### Antipsychotics

Many kinds of atypical antipsychotics have been confirmed effective in the treatment of children with BD. The review by Gentiles showed that short-term treatment with aripiprazole, olanzapine, and risperidone has been confirmed to be effective against manic episodes in adolescents with BD, although each drug has its own safety problems (73). Findling et al. (74) administered quetiapine (150–300 mg/day) to children with BD for 8 weeks and discovered no differences from the control group, with 9.3% having increased triglycerides and 4.7% with TSH levels, all of which indicated that the 8-week quetiapine treatment had no prominent effects but was safe and well tolerated. Barzman et al. (65) administered quetiapine (400–600 mg/day) to children comorbid with disruptive behavior disorder for 28 days and confirmed that it had an effect on impulsive and responsive aggressive behaviors, which could be considered as a kind of monotherapy method.

Findling et al. (75) administered aripiprazole (10 and 30 mg/day) to children with BD in an acute mania or a mixed symptom phase for 4 weeks, with response rates (≥50% reduction in YMRS total score) of 44.8 and 63.6%, respectively, and a response rate of 26.1% for the placebo. The most frequent side effects were headache, drowsiness, and extrapyramidal side effects (75, 76). Youngstrom et al. (56) also administered aripiprazole (10 and 30 mg/day) for 4 weeks, with response rates of 45 and 64%, respectively, and a response rate of 25.5% for the placebo. A meta-analysis by Meduri et al. (77) indicated that aripiprazole was effective against symptoms of pediatric BD after an acute 3-week therapy and did not lead to hyperprolactinemia and that the rate of extrapyramidal side effects was 24%, the rate of sedation was 23%, and the rate of other side effects was <20%. Findling et al. (76) administered aripiprazole (10 and 30 mg/day) to children with BD type I for 30 weeks and discovered a significant improvement in both treatment groups, with response rates of 58.7 and 64.8%, respectively, and a response rate of 29.7% for the placebo. The data indicate that aripiprazole is effective not only in the acute phase but also after 30 weeks of the treatment.

Kato et al. (78) showed that daily administration of lurasidone at 20–60 mg was more efficient than 80–120 mg and could significantly improve depression symptoms and functional impairment in adult patients with BD type I, with side effects that most frequently included akathisia and nausea, having little effect on weight, blood glucose levels, and lipids, similar rate of appearance of mania after treatment (3.8%) to the placebo group (2.3%). DelBello et al. (79) administered lurasidone (20–80 mg) to children with BD type I during a depressive for 6 weeks and found that 59.5% showed improvement in depression symptoms, anxiety, life quality, and total function, with side effects including nausea and drowsiness. However, few effects were found regarding weight and metabolism (79). Finally, the appearance of mania after treatment (1.7%) was similar to that of the placebo group (2.3%) (79). The data indicate that lurasidone is an effective treatment method for both adult patients and pediatric and adolescent patients with BD.

Joshi et al. (80) found that administration of paliperidone (3–6 mg) was significantly effective in treating adolescent BD symptoms, including elevated mood, irritability, and psychotic episodes, and was also effective for ADHD but was ineffective against depression, 73% appeared at least 30% reduction of mania scores in YMRS, 47% appeared improvement of ADHD symptom, the most frequently appeared side effects were low energy, cold, infection, allergic symptom, increased appetite, and headache, although paliperidone had no effect on the cardiovascular system but increased weight.

Pavuluri et al. (81) administered risperidone (0.5–2 mg/day) or divalproex sodium (60–120 μg/ml) to children with BD for 6 weeks and found that the risperidone group had quicker improvement of mania symptoms but total improvement and safety were similar between the two groups. Further, the response rates to YMRS were 78.1 and 45.5%, respectively, the remission rates were 62.5 and 33.3%, respectively, and risperidone group presented better improvement in CDRS-R scores (81). Finally, the dropout rate of the divalproex sodium group was higher, which might be due to increased irritability (81). West et al. (82) administered risperidone and divalproex sodium therapy to patients with BD comorbid with a disruptive behavior disorder and found more prominent improvement of mania symptoms in the risperidone group but found similar improvement for patients without disruptive behavior disorder. Further, patients without disruptive behavior disorder showed better improvement in the total function, which was more and more significant as the therapy progressed (82). Finally, the improvement of total function of patients with a high level of aggressivity was lower than those with a low level of aggressivity (82).

Findling et al. (83) conducted amoxapine (a second-generation atypical antipsychotic that is 5-HT, dopamine, NE, and histamine antagonist) therapy (2.5, 5, and 10-mg bid) and found prominent improvement in YMRS scores in patients receiving each dosage (42–54%) compared with the placebo group (28%), with over 5% suffering from drowsiness, sedation, oral hypoesthesia, and numbness. Further, increased appetite was apparent, 8–12% gained weight, and blood glucose and lipids also increased compared with the placebo group (83). Findling et al. administered amoxapine (2.5–10-mg bid) to children with BD type I in acute mania or mixed episodes for 3 weeks then continued treatment for an additional 50 weeks and found, after 26 weeks of treatment, that 79.2% showed a 50% reduction in YMRS scores, 83.2% had side effects including drowsiness/sedation/somnolence (42.4%) and oral hypoesthesia/ dysgeusia (7.5%), and 34.8% showed significantly increased weight gain (growing rate ≥ 7%) (84). Taken together, the data indicate that amoxapine is well tolerated at <50 weeks for children with BD type I (84).

### Comparison

A meta-analysis by Yee et al. (85) indicated that, for drug treatment of adolescent BD, clinical response rates of drug therapy (66.8%) were not significantly higher than those for the placebo (60.6%), but for no recurrence rates, drug therapy (56.7%) was significantly higher than that of the placebo (43.4%). Further, the efficiency order of drug combination (82.7%) was better than anticonvulsant (53.2%), followed by lithium (51.1%), which was better than antipsychotics (50.1%) (85). The authors also indicated that the appearance side effects were 5.5%−28.5%, which mostly included cognitive decline (28.5%), increased weight (28%), gastrointestinal symptoms, such as nausea and vomiting (25%), and epistaxis/suicidal ideation/diarrhea (5.5–6.6%) (85). The order of appearance rate of side effects from the highest to the lowest was lithium (23.9%), drug combination (23.4%), antipsychotics (20%), and anticonvulsant (85).

A review by Bailly (86) demonstrated that antipsychotics were more effective against mania or mixed episodes than mood stabilizers, which resulted in gastrointestinal and neural symptoms, whereas for antipsychotics, increased weight and sedation were commonly experienced. Joshi et al. (80) compared paliperidone treatment with aripiprazole, ziprasidone, olanzapine, risperidone, and quetiapine treatments and found that patients on paliperidone and aripiprazole showed the best improvement in YMRS scores, whereas those on ziprasidone and olanzapine showed the least improvement. The data also indicated that ziprasidone treatment resulted in the largest reduction of CDRS scores and that risperidone treatment resulted in the second-largest reduction of CDRS scores; however, there was no improvement in the brief psychiatric rating scale (BPRS) or ADHD–rank scale (ADHD–RS) scores (80). With regard to weight gain, paliperidone treatment was similar to atypical antipsychotics; however, olanzapine treatment resulted in significant increases in weight, whereas ziprasidone treatment resulted in the least (80). Correll (87) administered lithium or antiepileptic drugs, or the combination of both, second-generation atypical antipsychotics singly or in combination with lithium or divalproex sodium to children with BD and found significantly decreased weight gain in following treatment with topiramate or aripiprazole, whereas in treatment paradigms <12 weeks increased weight gain was evident in those using the second-generation atypical antipsychotics in combination with mood stabilizers than using mood stabilizer singly or the combination of mood stabilizer. Finally, the treatment with antipsychotics resulted in little rapid change in blood glucose or lipid levels (87). Henin et al. (88) indicated that the use of mood stabilizers resulted in poorer processing speeds and working memory than without treatment but that the use of antipsychotics did not affect cognitive impairment.

Scheffer et al. used a therapy strategy for the treatment of refractory pediatric and adolescent BD where the first step was to withdraw unstable factors such as antidepressants, GABA receptor antagonists, and stimulants; the second step was to optimize anti-mania drugs, and the third step was to use a limited number of mood stabilizers. After 6 months of treatment, 75.8% of refractory patients had a significant and long-lasting reduction in mania scores (89).

## Psychotherapy

### Family-Focused Treatment

FFT has been confirmed to be effective against pediatric and adolescent BD. Miklowitz et al. (90) conducted FFT and enhanced care (EC) as an adjunct to pharmacological treatment for children with BD and found that the two methods had similar effects on recovery time, recurrence, and proportion of weeks of illness but that patients administered FFT had less severe mania symptoms. Miklowitz et al. (91) also indicated that FFT and EC had similar effects on total recovery time, with 96.7% of patients after FFT meeting the recovery criteria of having a 4-week recovery period for mania and hypomania, whereas 100% of patients after EC met this criterion, but both treatments appeared a prominent improvement on depression symptom; FFT showed more rapid recovery, larger reduction of mood severity scores, and less time in acute depression phase but no advantage in the mania phase, which reached better recovery of symptom and function for adolescents, so FFT needs to be used in combination with means of intervention prone to mania state. O'Donnell et al. (92) showed that, for early-onset adolescent BD, the impairment of family function was related to more severe depression and mania symptoms, earlier recurrence, and increased suicidal behavior and that the adolescent-reported family cohesion increased after FFT but that, in terms of improvement in family conflicts, FFT and EC had similar outcomes. Moreover, for those children with high-risk BD and adolescents (children with prominent depression and mania symptoms, parents having mood disorders, and at least one parent showing high levels of criticism), FFT was confirmed to be effective and the interval time of depression was prolonged after 4-months of FFT (93). Meanwhile, after FFT, suicidal ideation significantly decreased, the interval time without suicidal behaviors was prolonged, and children-reported family conflicts decided the efficiency of FFT to suicidal ideation (94), clinically-related depression scores, and the frequency of perceived criticism from parents were also reduced (95). For high-risk children, FFT may therefore improve not only depression, hypomania, and psycho–social scores but also YMRS and CDRS scores (96).

Weintraub et al. (97) divided mood symptom trajectories of adolescent BD patients after FFT into the following four types: Apparent euthymia (initially responded to treatment well and maintained euthymia), accounting for 29.9% of cases; morbid but apparently improved (poor initial treatment effect but later improved and then maintained euthymia), accounting for 11.1%; mild euthymia (improved initially but symptoms reappeared and residual symptoms remained), accounting for 26.4%; and morbid with mild improvement (had severe symptoms initially and presented with mild euthymia after treatment), accounting for 32.6%; participants in these four groups spent, respectively, 77.7, 53.6, 44.1, and 18.6% of the follow-up weeks in euthymia states. Groups 1 and 2 showed fewer severe depression symptoms at baseline than Groups 3 and 4, and Groups 1 and 2 also showed less suicidal ideation, which meant that the prognosis of BD with more severe depression symptoms and suicidal ideation was poorer (97). Meanwhile, life stage and the level of therapy at baseline also predicted the division whereby patients in Groups 1 and 2 presented with higher life quality scores than patients in Group 4; patients in Group 2 presented with the highest family happiness levels at baseline and higher school happiness than patients in Groups 3 and 4, which indicated that life quality was related to mood symptoms of patients with BD (97). Weintraub et al. (98) documented three types of mood trajectories in children at risk of conversion to BD after FFT with 32.5% having apparent improvement, 16.7% mild residue symptoms, and 50.8% predominantly symptomatic course. In particular, patients in Group 3 had more severe depression symptoms at baseline, suicidal ideation, and anxiety severity, which indicated that more severe depression, anxiety, and suicidal ideation as predictors of poor prognosis, which might be used as individual therapies (98).

### Cognitive Behavioral Therapy

Feeny et al. (99) assessed patients with BD in the age range of 10–17-years and conducted CBT as an adjunct to pharmacological treatment, with parents reporting fewer depression and mania symptoms after treatment, which meant that CBT was also an effective method to BD.

### Interpersonal and Social Rhythm Therapy

Porter et al. (100) conducted IPSRT or unspecific clinical supportive treatment as an adjunct to pharmacological treatment for the adolescent patients with BD and showed that patients had prominent improvement in total cognitive composite scores and executive function and mental motor speed. Further, those with low baseline scores presented with better improvement, but no differences were found between the two kinds of treatments (100). Hlastala et al. (101) indicated that IPSRT improved children-reported satisfaction, with significant reductions in mania, depression, and general psychiatric symptoms after a 20-week treatment. Indeed, 75% had a ≥50% reduction in BPRS, 67% had a reduction in average depression and mania scores using the mania rating scale, and 53.2% had a reduction in the Beck Depression Inventory scores, with a significant improvement in total function (101).

### Child- and Family-Focused Cognitive-Behavioral Therapy

Child- and family-focused cognitive-behavioral therapy (CFF–CBT) has also been confirmed to be effective. Weinstein et al. (102) compared CFF–CBT with normally enhanced non-structural psychotherapy and discovered significant improvement of depression symptoms in those with higher levels of depression at baseline in low-income and highly cohesive families; for mania symptoms, those with low levels of depression at baseline and higher levels of self-esteem showed worse response to normal psychotherapy than CFF–CBT, and these differences were all unrelated to age, gender, mania level at baseline, comorbidity, and suicidal ideation. West et al. (103) stated that, after CFF–CBT, children with BD showed better engagement, less likely to drop out, higher satisfaction, significant reduction of parent-reported mania and depression symptoms, and a higher improvement of total function during the follow-up. MacPherson et al. (104) indicated that, after CFF–CBT, the improvement of mania, depression, and total function was better; meanwhile parenting skills, coping styles, family flexibility, and positive family restructuring also improved, all of which were related to the improvement of mood and function of children with BD.

### Other Psychotherapy Methods

Brief motivational intervention (BMI) and dialectical behavior therapy have also been confirmed to be effective against BD. Goldstein et al. (105) conducted BMI as an adjunct to pharmacological treatment, with results indicating that the compliance to drug treatment increased by 1% per month in groups incorporating BMI, and the compliance to drug treatment decreased by 5% per month in groups with drug treatment only. Further, poor compliance predicted an increased possibility of depression or mania symptoms in the next weeks whereas patients with compliance of over 60% had 3.6 times less likely to appear with depression symptoms in the next 2 weeks and 2.7 times less likely to appear mood symptom (105). Goldstein et al. (106) confirmed that dialectical behavior therapy patients were satisfied with the treatment and had high therapy satisfaction and apparent improvement of suicide ideation, self-injury, mood dysfunction, and depression symptoms after treatment.

A psychological intervention aimed at clinical workers in a psychiatric department has also been shown to effectively increase the diagnostic accuracy rate. Jenkins et al. (107) conducted cognitive debiasing intervention (educated some cognitive pitfalls like base-rate neglect and search satisfying and taught correct strategies like mnemonics, Bayesian tools) to clinical practitioners with results indicating higher total accuracy of judgment to diagnose pediatric and adolescent BD and fewer decision-making mistakes after treatment. Overall, cognitive debiasing intervention helped to identify the mismatch problem between typical thinking habits when confronted with children with BD and disease traits (107).

## Conclusion

In conclusion, BD and its subtypes still have high morbidity. Although high rates of BD first manifest during childhood or at puberty, children with BD do not readily receive therapy in a timely manner which might be related to the varieties of clinical magnification of pediatric and adolescent BD. Pediatric and adolescent patients with BD present with more irritability than mood disorders and a higher risk of suicide accompanied with behavioral problems, such as increased activity, attention deficits, and aggressiveness; therefore, clear distinctions between BD, ADHD, and several other kinds of behavioral disorders are important. Meanwhile, pediatric and adolescent patients with BD have high comorbidity with many kinds of diseases, which increases the difficulties of diagnosis and therapy. The use of scales, such as YMRS and GBI-M10, can be beneficial in the correct diagnosis and aid in the proper intervention. In terms of clinical therapy, considering the specificity of children and adolescents, the safety of drugs needs to be considered. To date, several drugs such as mood stabilizers, quetiapine, aripiprazole, lurasidone, paliperidone, and risperidone, as well as several types of psychotherapy, including FFT, CBT, IPSRT, and CFF–CBT, have been confirmed to be effective. However, at this stage, the pathological mechanism of BD is still not clear and needs to be studied further.

## Author Contributions

GZ designed and revised the paper. LL wrote the paper. MM and XZ collected references. All authors contributed to the article and approved the submitted version.

## Funding

This work was supported by grants awarded by the Major Project of the Department of Science and Technology of Liaoning Province, China (2019JH8/10300019).

## Conflict of Interest

The authors declare that the research was conducted in the absence of any commercial or financial relationships that could be construed as a potential conflict of interest.

## Publisher's Note

All claims expressed in this article are solely those of the authors and do not necessarily represent those of their affiliated organizations, or those of the publisher, the editors and the reviewers. Any product that may be evaluated in this article, or claim that may be made by its manufacturer, is not guaranteed or endorsed by the publisher.
